# Advances in the Study of circRNAs in Hematological Malignancies

**DOI:** 10.3389/fonc.2022.900374

**Published:** 2022-06-20

**Authors:** Jingyi Du, Feiyu Jia, Lijuan Wang

**Affiliations:** ^1^School of Clinical Medicine, Shandong First Medical University & Shandong Academy of Medical Sciences, Jinan, China; ^2^Central Laboratory, Linyi People’s Hospital, Linyi, China; ^3^Department of Education and Teaching, Linyi People’s Hospital, Linyi, China; ^4^Linyi Key Laboratory of Tumor Biology, Linyi, China

**Keywords:** circRNAs, hematological malignancies, biomarkers, oncogenes, proliferation, apoptosis

## Abstract

Circular RNAs (circRNAs) are non–protein-coding RNAs that have a circular structure and do not possess a 5` cap or 3` poly-A tail. Their structure is more stable than that of linear RNAs, and they are difficult to deform *via* hydrolysis. Advancements in measurement technology such as RNA sequencing have enabled the detection of circRNAs in various eukaryotes in both *in vitro* and *in vivo* studies. The main function of circRNAs involves sponging of microRNAs (MiRNAs) and interaction with proteins associated with physiological and pathological processes, while some circRNAs are involved in translation. circRNAs act as tumor suppressors or oncogenes during the development of many tumors and are emerging as new diagnostic and prognostic biomarkers. They also affect resistance to certain chemotherapy drugs such as imatinib. The objective of this review is to investigate the expression and clinical significance of circRNAs in hematological malignancies. We will also explore the effect of circRNAs on proliferation and apoptosis in hematological malignancy cells and their possible use as biomarkers or targets to determine prognoses. The current literature indicates that circRNAs may provide new therapeutic strategies for patients with hematologic malignancies.

## Introduction

Circular RNAs (circRNAs) are noncoding RNAs formed by reverse splicing, with a downstream splice donor covalently linked to an upstream splice acceptor (1). circRNAs are classified into four types: exonic circRNAs (ecircRNAs), containing only postsplicing exons; circular intron RNAs (ciRNAs), formed from introns; exon-intron circRNAs (EIciRNAs), which are exon–intron circRNAs; and tRNA intronic circular RNAs (tricRNAs) (2, 3). ecircRNAs are abundant among mammalian transcripts, display strong conservation, and are highly expressive (4). ciRNAs are frequently detected in the nucleus and positively regulate parental gene expression (5). EIciRNAs are mainly localized in the nucleus and regulate the expression of parental genes in *cis* through positive feedback (6). The tricRNAs are stable and abundant, with typical characteristics of postnatal transcripts (7). Different isoforms of circRNAs can be produced from a single gene, such as CircSTAU2a and CircSTAU2b (8)

Currently, several mechanisms of circRNA biogenesis are known. Intron pairing-promoted cyclization (9), spliceosome-dependent lasso-driven cyclization (10), and facilitation of the 3’-end to 5’-end ligation of circRNAs by RNA binding proteins (RBPs) (11) are the most common biogenesis mechanisms of ecircRNAs and EIciRNAs. Furthermore, ciRNA formation is dependent on the intron, a GU-rich sequence in the lasso at the 5’ end, and an AC-rich sequence at the branch point (5). tricRNAs result from the intron ends generated by the pre-tRNA cleavage (12).

In 1976, circRNA was first recognized in plant viroids (13), and in 1979, circRNAs were first detected in human HeLa cells using electron microscopy (14). Subsequently, circRNAs were found in hepatitis D virus, which was the first instance that circRNAs were detected in an animal virus (15). Earlier, studies were not conducted on circRNAs because they were considered as minor products of RNA missplicing (16, 17). With the development of analytical techniques such as RNA sequencing and informatics tools, circRNAs have been detected in various eukaryotes (e.g., mice), as well as in human plasma and saliva (8, 10, 18–20).

Since the creation of the first genome-wide circRNA atlas in 2012 (21), various circRNA identification tools such as Find-CIRC, MapSplice, and CircRNAFisher have been constructed, and several circRNA databases including CircBase, Circpedia, and CircRNADb have been established (18). In addition, Dahl et al. obtained differential expression profiles that can distinguish between different B-cell malignancies using nano-string technology (22); and Issah et al. established the expression profile of N6-methyladenosine (m6A) circRNAs in AML patients by microarray analysis (23). Using these tools, the preliminary identification, characterization, and annotation of circRNAs can be achieved (18). These technologies have facilitated the advancement of circRNA research (18), and more than 10,000 human CircRNAs have been identified till date (8).

CircRNAs have a stable circular structure that lacks a 5` cap and 3` poly-A tail; therefore, it is difficult to deform circRNAs *via* hydrolysis (24). Meanwhile, the sequence of circRNAs is more strongly conserved and extensive than the corresponding linear RNAs. It has also been suggested in many studies that circRNAs display high specificity across various tissues (17). Furthermore, circRNAs can promote the transcription of parental genes by acting in *cis-* or *trans-* (25).

The main functions of circRNAs are as follows. Firstly, circRNAs are considered microRNA (miRNA) sponges (26). Mir-7 is one of the most widely known miRNAs as a target of circRNAs, both suppressing and inducing tumor progression (17). circRNA sponge for miR-7 (CIRS-7, also called CDR1as) has 74 conserved binding sites against mir-7 (4); the binding sites are predominantly located in the brain of placental mammals (27, 28), wherein they inhibit mir-7 and upregulate the expression of genes such as epidermal growth factor receptor (17). Secondly, circRNAs interact with proteins to influence protein expression, biogenesis, and pathophysiological processes (29). RBPs are a class of proteins that interact with RNA to regulate RNA maturation, translation, and other functions (29, 30). CircRNAs may bind to regulated RBPs and affect the expression of their target miRNAs (29). Next, even though the 5’ cap and 3’ poly-A tail required for mRNA translation are absent in circRNAs, they are translatable at sites such as the internal ribosome entry site (IRES) and m6A modification in their sequences, and many circRNAs have now been shown to produce proteins by translation (17, 31). The modification of circRNA by m6A contributes to the translation of circRNA (32). Recently, circRNAs have been found to play important roles in the proliferation and apoptosis of various tumors (33–36). The chromosome 9 and 22 translocations in chronic myelogenous leukemia (CML) produce the PML-RARα gene; similar chromosomal translocations in tumors are reported to produce fusion-circular RNAs (f-circRNAs) and facilitate the progression of diseases such as leukemia (37, 38). Moreover, circRNAs show potential as new therapeutic targets for and biomarkers of diseases (39). circPVT1 is considered to be a prognostic marker for gastric cancer (40). In recent years, increasing evidence has indicated that circRNAs play important roles in hematological tumor development and progression. These tumors have high circRNA content, are not easily degraded and are widely present in various body fluids; therefore, exploring circRNAs associated with hematologic malignancies as biomarkers or targets will create new possibilities for diagnosis, prognosis, and effective treatment. In this review, we summarize the expression of different types of circRNAs in hematological malignancies, describe their role in tumor cell growth and apoptosis. (**Table 1**).

**Table 1:** Some abnormal circRNAs with confirmed functions in the universal category of hematological malignancies (↑: up-regulated;↓:down-regulated).

CircRNA	Disease	Expression	Clinical significance	Reference
Circ-00004136	AML	↑	Promote cell proliferation	(41)
Circ-RNF13	AML	↑	Inhibited the proliferation, migration, and invasion of AML cells	(42)
Circ-VIM	AML	↑	An independent poor prognostic factor for AML patients	(43)
Circ-RAD18	AML	↑	Promote proliferation and accelerate tumor progression	(44)
Circ-PTK2	AML	↑	Accelerate the tumor progression and inhibit apoptosis	(45)
Hsa-circ-0000370	AML	↑	Accelerate tumor cell development	(46)
Hsa-circ-0121582	AML	↓	Inhibiting tumor progression	(47)
Circ-0002232	AML	↓	A potential biomarker for AML	(48)
Circ_0000745	ALL	↑	Promote cell proliferation	(49)
Circ_PVT1	ALL	↑	Inhibit the tumor apoptosis and accelerate progression	(50)
Circ_100053	CML	↑	Increased imatinib resistance	(51)
Circ_0080145	CML	↑	Increased imatinib resistance	(52)
Circ_0009910	CML	↑	Increased imatinib resistance	(53)
Circ_CBFB	CLL	↑	Accelerate the proliferation of CLL cells and inhibit apoptosis	(54)
Circ_COX2	CLL	↑	Accelerate the tumor apoptosis and inhibit progression	(55)
Circ-APC	DLBCL	↓	Biomaker in diagnosis and prognosis	(56)
CircCFL1	DLBCL	↑	Interfere the migration and proliferation	(57)
circ-LAMP1	T-LBL	↑	Promote proliferation and inhibit apoptosis	(58)
Circ_0000142	MM	↑	promotes MM progression	(59)
CircRNA ITCH	MM	↓	Increase bortezomib sensitivity	(60)
Hsa_circ_0069767	MM	↑	Decrease migration and invasion capacities	(61)
Circ-SMARCA5	MM	↓	Inhibit the progress of MM	(62)
Circ_0000190	MM	↓	Inhibit the progression of MM	(63)
Circ_0007841	MM	↑	promotes the progression of MM	(64, 65)

Acute lymphoblastic leukemia, ALL; Acute myelogenous leukemia,AML; Chronic myelogenous leukemia,CML; Chronic lymphocytic leukemia, CLL; Circ-chromodomain Y like, CircCDYL; Circ‐vimentin, Circ-VIM; Diffuse large B cell lymphoma, DLBCL; T-cell lymphoblastic lymphoma, T-LBL; Multiple myeloma, MM.

## CircRNAs and Leukemia

AML is a disease caused by malignant proliferation of myeloid cells in the bone marrow (45).Acute myelogenous leukemia (AML) is the most prevalent acute leukemia in adults (66). Although the diagnosis and treatment strategies of AML have been rapidly developed, the prognosis remains poor (44). With the advancement of analytical techniques, many circRNAs have been identified to be abnormally expressed in AML. In a study screening 273 circRNAs upregulated in AML, circ-00004136 was found to be significantly upregulated in pediatric AML, promoting leukemic cell proliferation *via* miR-142 sponging (41). circ-RNF13 was significantly upregulated in adult patients with AML and it inhibited AML cell proliferation, migration, and invasion by regulating miRNA-1224-5p expression (42). miRNA-1224-5p overexpression promotes early apoptosis of AML cells and arrests the cell cycle in the G1 phase (42). circ-vimentin expression has been found to be significantly upregulated and positively correlated with leukocyte counts and French–American–British (FAB) classifications. Furthermore, circ-vimentin overexpression is considered an independent and poor prognostic factor for significantly shorter overall survival (OS) and leukemia-free survival (LFS) in patients with AML (43). circ-RAD18 was found to be highly expressed in patients with AML, promotes AML cell proliferation, and accelerates tumor progression *via* miR-206/*protein kinase CAMP-activated catalytic subunit beta (PRKACB)* (44). *PRKACB* participates in various biological processes, including cell replication, gene transcription, and metabolism (44, 67). circRAD18 directly inhibits miR-206, whereas miR-206 inhibits *PRKACB* (44). circ-PTK2 affects the miR-330-5p/forkhead box M1(FOXM1) axis by binding to miR-330-5p, accelerating tumor progression, and inhibiting tumor apoptosis (45). circ-0009910, circ-0058058, and circ-DLEU2 are also overexpressed in AML, and their expression is negatively correlated with patient prognosis (67–69). AML patients with FMS-like tyrosine kinase 3 receptor (FLT3) mutations tend to have shorter survival rates and are more likely to experience relapse than patients without these mutations (70). hsa-circ-0000370 expression was found to significantly increase in patients who had AML with FLT3 internal tandem duplication and promoted tumor development by inhibiting miR-1299 expression (46). hsa-circ-100290 and hsa-circ-0079480 expression was also found to be elevated in AML, leading to the promotion of AML *via* the inhibition of miR-203 and miR-654-3p expression, respectively; hsa-circ-100290 and hsa-circ-0079480 knockdown induced apoptosis in AML cells (71, 72).

circRNA expression is downregulated in patients with AML. Lower circ-0002232 expression is correlated with increased patient survival and decreased phosphatase and tensin homolog deleted on chromosome 10(PTEN) expression (48). circ-0002232 might influence PTEN expression and AML progression *via* miR-92a-3p sponging (48). The expression of hsa-circ-0121582, a robust circRNA reverse-spliced from exon 1 to exon 7 in GSK3β, decreases in AML and inhibits tumor progression by binding to miR-224 and TET1 *via* the Wnt/β-catenin signaling pathway (47). Regardless of their expression in AML, circRNAs are expected to be developed as novel biomarkers or therapeutic targets for the diagnosis and treatment of AML, involved in assessing patient prognosis, early risk stratification, likelihood of recurrence, and treatment by targeting circRNA-miRNAs and related signaling pathway networks.

Acute lymphoblastic leukemia (ALL) is a malignant neoplastic disease in which lymphocytes proliferate abnormally in the bone marrow (73). ALL is more frequent in children, with adequate prognosis, while its prognosis in adults remains inadequate (49). circRNAs have the potential to serve as novel therapeutic targets to improve the prognosis of ALL patients. circ-0000745 and circ-PVT1 expression was found to be elevated in ALL (49, 50). circ-0000745 overexpression activates the ERK pathway, thereby promoting cell proliferation (49). In most cancers, MYC expression is elevated; high MYC expression indicates poor prognosis in patients with ALL (74). c-MYC is a member of the MYC family (75). The gene on chromosome 8q24.21 is named human *plasmacytoma variant translocation* 1 (*PVT1*), which is located 54 kb far downstream of MYC. circPVT1 is generated by cyclization of exon 2 of *PVT1* (16, 76). High circPVT1 expression in ALL leads to the inhibition of c-MYC expression, in turn inhibited the apoptosis of ALL cells and promotion of cell proliferation (50). If the proliferation of ALL cells can be inhibited by targeting these circRNAs, then it is possible to improve the prognosis of ALL patients.

CML is a hematological malignancy caused by reciprocal translocation between the long arms of chromosomes 9 and 22 (77, 78). This translocation also creates the breakpoint cluster region (BCR) and Abelson (ABL) genes (77, 79). Imatinib is a first-line drug for CML (80). Drug resistance is a major obstacle to CML treatment (81). High circ-100053 and circ-0080145 levels are associated with imatinib resistance in CML (51, 52). Patients with high circ-100053 levels are more likely to develop resistance to imatinib than patients with low circ-100053 levels, thereby leading to worse prognosis (51). Moreover, circ-100053 is related to the BCR/ABL mutation status (51). circ-0080145 expression was found to be higher in patients who were resistant to imatinib than imatinib-sensitive patients. circ-0080145 knockdown increased imatinib-sensitivity of patients with CML, inhibited imatinib resistance and cell proliferation, and induced apoptosis of imatinib-resistant CML cells *via* miR-326 (circ_0080145 target) and protein alpha 1 (miR-326 target gene) (52). hsa-circ-0080145 was found to be highly expressed in CML cells with high proliferative capacity; furthermore, hsa-circ-0080145 directly inhibited miR296 (82). circBA9.3, a circRNA derived from BCR-ABL1, has been detected in some imatinib-resistant patients (83). circBA9.3 was found to be positively correlated with BCR-ABL1 expression, and the presence of BCR-ABL1 indicated poor prognosis. circBA9.3 has been reported to inhibit CML cell apoptosis and promote BCR-ABL1 protein expression (83). circ-0009910 accelerates CML resistance to imatinib *via* the miR-34a-5p/ULK1 axis (53). These findings suggest that circRNA has a significant impact on imatinib resistance, and may therefore serve as a therapeutic target for imatinib-resistant CML and potential biomarker for the diagnosis of CML.

Chronic lymphocytic leukemia (CLL) involves the malignant proliferation of CD5-positive monoclonal B lymphocytes (84, 85). circ-CBFB was found to be significantly overexpressed in CLL, which accelerated CLL cell proliferation and inhibited CLL cell apoptosis (54). circ-CBFB promotes FZD3 expression by inhibiting miR-607 *via* the activation of the Wnt/β-catenin pathway and acceleration of CLL progression (54). High mc-COX2 (mitochondrial genome–derived circRNA) expression was reported to be inversely correlated with prognosis while decreased mc-COX2 expression was found to affect mitochondrial function, inhibit cell proliferation, and induce apoptosis (55). Plasma circ-RPL15 was also found to promote CLL progression (86).

**Figure 1:**
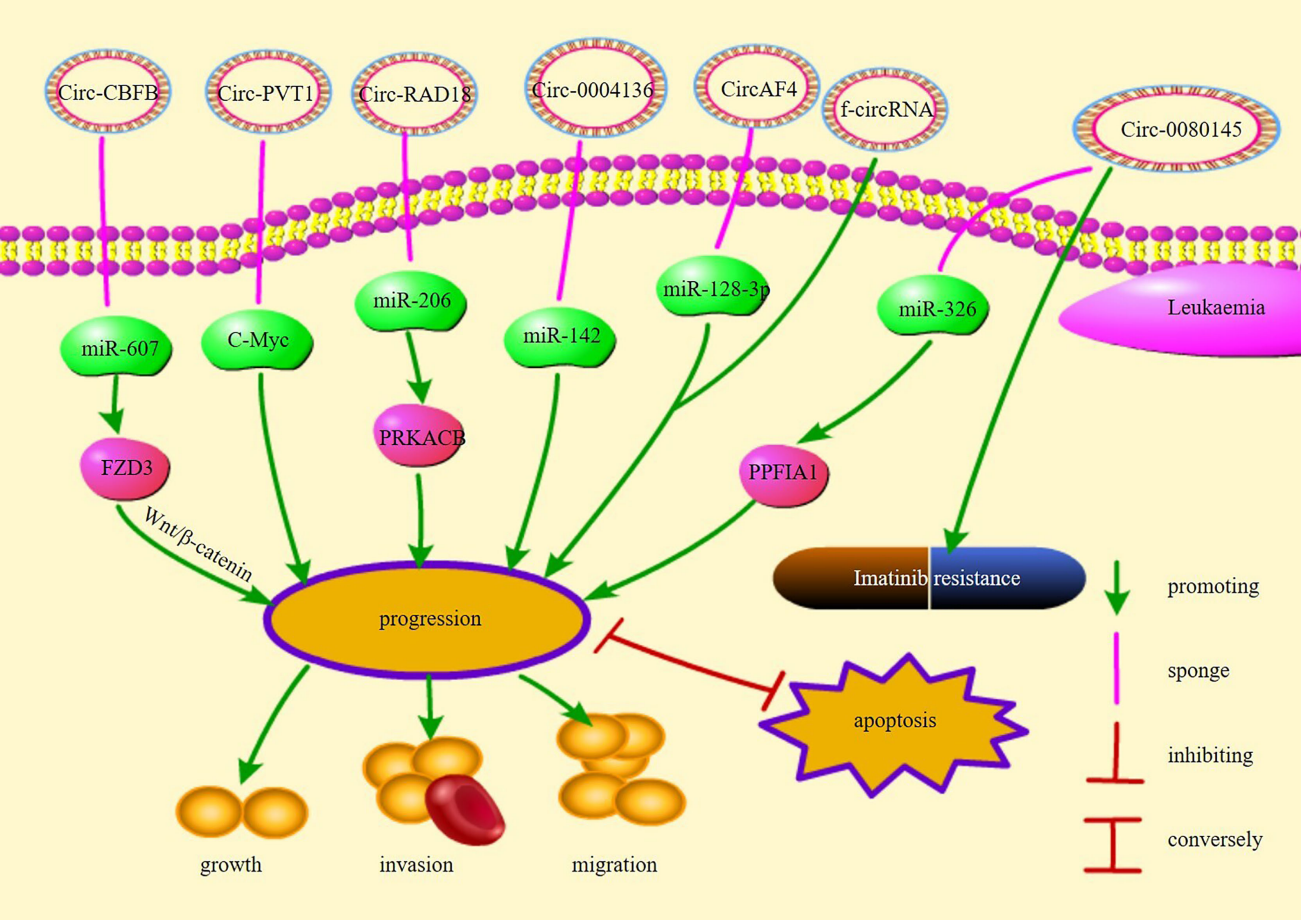
The regulatory mechanisms and their functions of circRNAs in leukemia.

Chromosomal translocations may result in oncogenic fusion genes involved in the development of many tumors and are an important cause of leukemia (87). Guarnerio et al. demonstrated that f-circRNAs produced by chromosomal translocations associated with leukemia promote proliferation and disease progression and maintain the viability of leukemic cells, thereby increasing resistance to chemotherapy and reducing treatment efficacy (87). In addition, further insights into the origin of fusion gene chaperone circRNAs (FP-circRNAs) revealed that these FP-circRNAs have the potential to function as oncogenes in chromosomal translocation leukemias (88). For example, CircAF4 found in MLL leukemia originates from the partner of the MLL fusion gene *AF4*, promotes leukemogenesis, and represses MLL-*AF4* gene expression through the sponge miR-128-3p (89). In summary, f-circRNAs and FP-CircRNAs may be considered a target for the diagnostic treatment of chromosomal translocation leukemia. **Figure 1** illustrates the pathways of action and functions of circRNAs in leukemia.

## CircRNAs and Lymphoma

Lyphomas may be classified into various types, and the clinical presentation, treatment options, and prognosis vary based on the classification (90). T-cell lymphoblastic lymphoma (T-LBL) is a malignant tumor that arises from malignant thymocyte proliferation, leading to massive infiltration of immature T cells in the mediastinum and in lymphoid organs (90). Recently, circ-LAMP1 was found to be overexpressed in T-LBL tissues. circ-LAMP1 was reported to indirectly regulate discoidin domain receptor tyrosine kinase 2 (DDR2) expression by repressing miR-615-5p and to directly promote DDR2 expression and inhibit T-LBL cell apoptosis *via* the miR-615-5p/DDR2 pathway (58).

**Figure 2:**
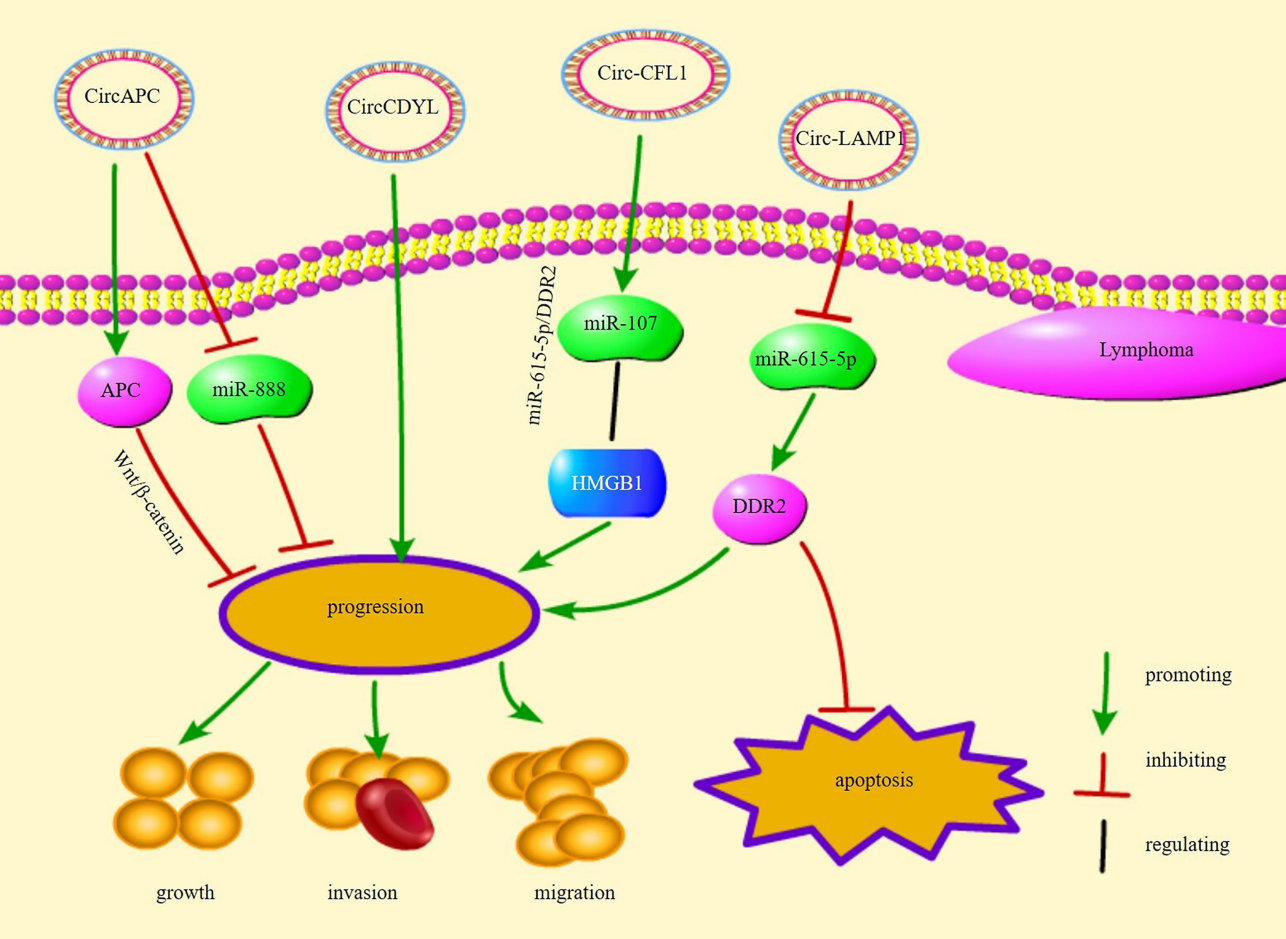
The regulatory mechanisms and their functions of circRNAs in lymphoma.

Diffuse large B-cell lymphoma (DLBCL) is the most common form of lymphoma in adults; approximately 30% of patients with DLBCL cannot be cured (91–93). It is a highly aggressive disease that originates in or outside the lymph nodes and is also caused by the transformation of other malignancies, such as CLL (92, 94, 95). circ-APC, a circRNA originating from the back-splicing of APC exon 7 to exon 14, sponges and decreases miR-888 expression or physically combines with the APC promoter to increase APC expression (56). circ-APC is a possible biomarker for DLBCL diagnosis and prognosis (56). APC inhibition decreases β-catenin aggregation in the nucleus, thereby suppressing the Wnt/β-catenin signaling pathway and inhibiting DLBCL growth (96). circCFL1 promotes the migration and proliferation of DLBCL cells (57). Using the dual-luciferase reporter gene system, it was confirmed that circCFL1 sponges its target gene miR-107 to reduce miR-107 expression, which binds to its target gene high mobility group box (HMGB)1 and reduces HMGB1 expression (57). HMGB1 enhances tumor invasion and metastasis and accelerates tumor growth (97). circCFL1 regulates the HMGB1 level *via* mir-107 and consequently promotes DLBCL cell migration and proliferation (57).

circ-chromodomain Y-like (circCDYL) is overexpressed in mantle cell lymphoma (MCL) and promotes MCL cell proliferation (98). Receiver operating characteristic curve analysis showed that circCDYL is a potential diagnostic biomarker for MCL (98). Overall, these circRNAs are involved in the proliferation, growth, and apoptosis of different types of lymphoma cells, implying that circRNAs are closely related to the progression of lymphoma and are potential therapeutic targets for lymphoma. **Figure 2** illustrates several circRNA pathways of action and their functions in lymphomas.

## CircRNAs and Multiple Myeloma

Multiple myeloma (MM) is the second most prevalent hematologic malignancy worldwide (99). It accounts for 1–2% of tumors worldwide and 2% cancer-related deaths (100). circRNAs play an important regulatory role in MM. Certain circRNAs are overexpressed in MM. Recently, it was shown that circ-CDYL is significantly overexpressed in both MM cells and plasma (101). circ-CDYL promotes the growth of MM cells by absorbing miR-1180 and increasing YAP expression (101). circ_0000142 was found to be overexpressed in patients with MM and was significantly correlated with higher International Staging System (ISS) and Durie-Salmon stages (59). circ_0000142 promoted MM cell proliferation, migration, and invasion and inhibited apoptosis by regulating miR-610/AKT3 expression (59). hsa-circRNA-101237 expression in the bone marrow is significantly upregulated in patients with MM, in MM cell lines, and in BTZ-resistant MM cells, especially those with recurrent/refractory disease (102). hsa-circRNA-101237 expression was found to significantly increase in patients who had MM with t (4, 14), t (14, 16), 13q14 deletion, P53 deletion, and 1q21 amplification; the hsa-circRNA-101237 expression was related to the prognosis, OS, and progression free survival (PFS) of patients with MM (102). circ_0007841 was overexpressed in MM cell lines and patients with MM and induced the activation of the PI3K/AKT signaling pathway *via* the miR-338-3p/bromodomain containing 4(BRD4) axis (64). circ_0007841 induced the cell cycle, cell growth, and metastasis and inhibited the apoptosis of MM cells *via* the miR-338-3p/BRD4 axis (64). The malignant behavior of MM cells is augmented by exosomes obtained from mesenchymal stromal cells (MSCs) *via* circ_0007841 (64). hsa-circ-0007841 was found to be overexpressed in MM drug-resistant cells and was associated with chromosomal aberrations, including 1q21, t (4:14), ATR, and IRF4 gene mutations (103, 104). hsa-circ-0007841 induces resistance in MM cells to doxorubicin by promoting ATP-binding cassette transporter G2 (ABCG2) (104). These studies suggest that overexpression of circRNAs promotes disease progression in MM, indicating their close association with its development, and nominate them as novel potential targets and biomarkers for the treatment of MM for diagnosis, and disease staging, among other uses.

Bortezomib (BTZ) is an effective drug for treating primary and relapsed MM (105). BTZ resistance remains a critical issue to overcome in the current MM treatment process (106). CircRNA itchy E3 ubiquitin protein ligase(circITCH) was found to decrease in the bone marrow of patients with MM, MM cell lines, and BTZ-resistant MM cells (60). Decreased circITCH expression is related to the poor prognosis of patients with MM (60). circITCH overexpression leads to the promotion of the sensitivity of BTZ-resistant MM cells to BTZ *via* the mir-615-3p/PRKCD pathway (60). circ_0007841 deletion reduces the resistance in MM cells to BTZ *via* the miR-129-5p/JAG1 axis (65). Therefore, targeting specific circRNAs may reduce the resistance of MM cells to BTZ and enhance the efficacy of the drug. In addition to BTZ, immunomodulatory drugs (IMids) such as lenalidomide and pomalidomide are crucial players in the treatment of MM (107). IMid-resistant cells affect the expression of circRNAs including CIRS-7, which is the most prominently affected circRNA. However, CIRS-7 knock-down does not reverse the drug sensitivity of MM cells. This suggests that CIRS-7 does not mediate the development of drug resistance in IMids (28).

Some circRNAs have been shown to have antitumor effects. circ-0069767 is overexpressed in MM, and its expression is correlated with longer PFS and OS (61). circ-0069767 overexpression inhibits the growth, migration, and invasion of MM cells and promotes apoptosis by sponging miR-636, which modulates KRAS expression (61). circ-MBYL2 was reported to decrease in the tissues and serum of patients with MM and was significantly related to advanced clinical stage and worse prognosis (108).Serum circ-MYBL2 analysis has been shown to be accurate for MM diagnosis (108). circ-MBYL2 overexpression inhibits the DNA synthesis in and cell cycle and growth of MM cells and decreases its viability by suppressing the binding of cyclin F to MYBL2, which inhibits the transcription of proliferation-related oncogenes (108). A study showed that circ_0000190 expression decreased but that of its target miR-767-5p increased in the bone marrow and peripheral blood of patients with MM (63). circ-0000190 suppressed cell cycle and growth and promoted the apoptosis of MM cells by regulating miR-767-5p/mitogen-activated protein kinase 4 (MAPK4) (63). Overexpression of Circ-0000190 arrests the cell cycle in G1 phase, and miR-767-5p promotes MM cell development by directly targeting MAPK4. Survival data analysis suggests that patients with high CIRC_0000190 expression have lower risk, and longer PFS and OS (63). circ-AMARCA5 expression has been found to decrease in MM and is negatively associated with beta-2-microglobulin (β2-MG) levels and ISS stage (62). circ-AMARCA5 overexpression was found to be related to improved complete response (CR), PFS, and OS and to suppress cell growth; meanwhile, it induced the apoptosis of MM cells by sponging miR-767-5p (62). In summary, patient prognosis may be improved by targeting circRNAs and their targets, and circRNAs display remarkable potential as biomarkers for MM.

Exosomes are secretory vesicles from the endosome (endosomal) with a diameter of about 40–160 nm (average diameter 100 nm) (109). They are widely distributed and abundant in body fluids (110) and can be secreted by most cells (111). Exosomes deliver nucleic acids including circRNAs, proteins, and other substances between cells, and regulate various pathophysiological activities (110, 112). CircDAP3, an exosomal CircRNA significantly decreased in essential thrombocythemia (ET), inhibited the conversion of K562 cells to megakaryocytes in *in vitro* studies, suggesting its possible involvement in the progression of ET (113). Peripheral neuropathy is the most prominent complication in MM (111), and is also one of the side effects of bortezomib (114). One of the circulating exosomal circRNAs exo-CircRNAs called ChR2:2744228-2744407+, which is upregulated in MM, has been found to potentially be involved in the induction of MM-induced peripheral neuropathy (111). Another serum exosomal CircRNA called CircMYC was shown to be significantly elevated in MM and suggested a poor prognosis. Expression was upregulated in relapsed refractory patients compared to primary patients, suggesting that it may be associated with high recurrence of MM. Additionally, expression was higher in BTZ-resistant patients compared to non-resistant patients, suggesting that CircMYC may be associated with BTZ resistance in patients (110). IgG MM interferes with a variety of cells in the tumor microenvironment by secreting CircHNRNPU *via* exosomes (115). Exosomal circ-ATP10A promotes angiogenesis in MM patients by targeting various miRNAs, such as hsa-miR-3620-3p, which is expected to improve the prognosis of patients (116). In summary, circRNAs secreted by exosomes can influence the course of MM, participate in the generation of MM complications, and interfere with the tumor microenvironment, providing a new entry point for the treatment of MM.

**Figure 3:**
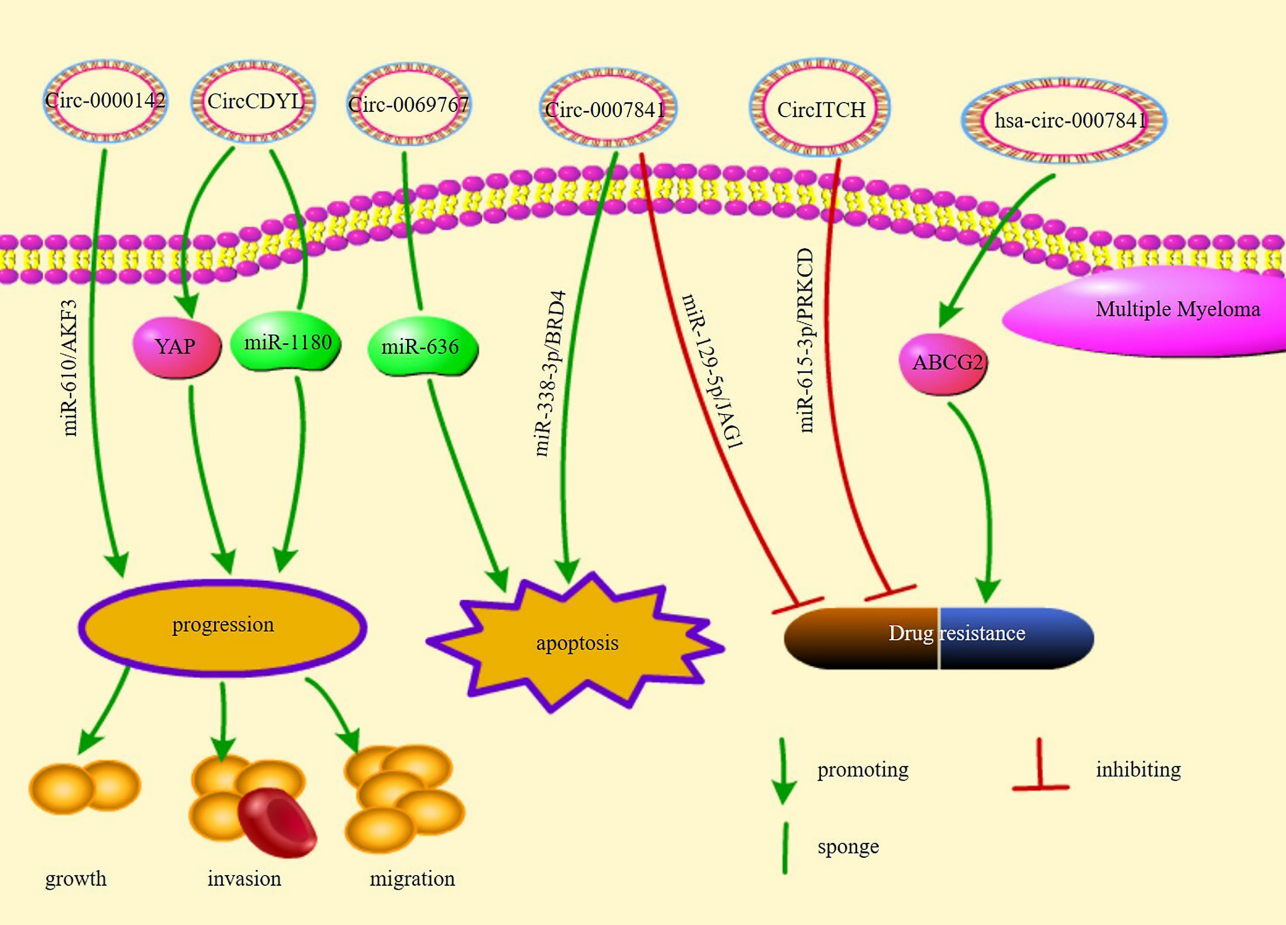
The regulatory mechanisms and their functions of circRNAs in multiple myeloma.

**Figure 3** illustrates the pathways of action and functions of circRNAs in MM.

## Discussion and Challenges

There is an increasing number of studies on circRNAs and hematologic malignancies. The expression of certain circRNAs, the interaction of many circRNAs with miRNAs, and the related signaling pathways are associated with growth, proliferation, invasion, and apoptosis of various hematological malignancies, and are also closely related to disease stage, OS, LFS, and PFS. In addition, the circRNAs participate in or manifest drug resistance, indicating their potential as therapeutic targets and biomarkers. Biomarkers are used for disease diagnosis, to determine disease staging, or to evaluate the safety and efficacy of new drugs or therapies in the target population (117). To become biomarkers or therapeutic targets, they must first have a high degree of specificity so that they can be targeted while reducing toxicity to normal cells; in addition, they must be sensitive and easy to detect and they must have a high degree of stability (118). Biomarkers and therapeutic targets need to undergo repeated screening, risk assessment, clinical trials, and prognostic analysis before they can be applied (117). CircRNAs in human blood (119) and other body fluids are abundant and easy to detect, indicating that CircRNAs are suitable as biomarkers or therapeutic targets. However, the specificity of circRNAs as diagnostic markers is still unclear, and their sensitivity as therapeutic targets is also unknown. Since a single circRNA may be aberrantly expressed in different diseases. For example, abnormal expression of circPVT1 can be detected in diseases such as gastric cancer and ALL (40, 50), so the diagnostic specificity of a single circRNA is insufficient. However, cumulative diagnosis may be achieved by combining multiple circRNAs or including corresponding target miRNAs, etc. Most of the current studies primarily focus on the interaction between circRNAs and miRNAs. Further mechanisms of action of circRNAs, as well as the case of multiple circRNAs that demonstrate abnormal expression in the same disease, require more investigation. Interactions between circRNAs are yet to be fully elucidated; a lack of sufficient clinical studies limit the applications these circRNAs. We have concluded the current clinical trials in **Table 2**.

**Table 2:** The clinical trials about circRNAs.

RNAs	Disease/condition	Status	Trial ID	Intervention/treatment
CircRNA	ALS	Recruiting	NCT05098340	Genetic: Quantitative Real-time polymerase chain reaction
AISRNA	AS/IS	Recruiting	NCT04175691	Genetic: Sequencing of circRNA/miRNA/lncRNA
EVTRNA	AS/IS/ET	Recruiting	NCT04230785	Genetic: Sequencing of circRNA/lncRNA/miRNA
CircRNA	ALI/ARDS	Recruiting	NCT03766204	
CircRNA/non-coding RNA	Pancreaticobiliary Cancers	Recruiting	NCT04584996	
CircRNA-Uck2	AMI	Unknown	NCT03170830	Diagnostic Test: the diagnosis value of cUck2 in AMI
circRNA from TEPs	Neuroendocrine Tumors	Recruiting	NCT04175691	Drug: Somatostatin analog; chemotherapy
CircRNA	Cardiac Arrest	Unknown	NCT02297776	
mRNA/circRNA/lncRNA	Young-COSMOS	Recruiting	NCT04864457	The alteration of RNA expression

Acute Ischemic Stroke, ALS; Circulating non-coding RNA in acute Ischemic stroke, AISRNA; Acute Stroke, AS; Ischemic Stroke, IS; Endovascular Treatment, ET; Acute Lung Injury, ALI;

Acute Respiratory Distress Syndrome, ARDS; Acute Myocardial Infarction, AMI; Young Adults Coronary Syndrome Patients, Young-COSMOS.

## Conclusion

In this review, we summarized the progress of circRNA research in hematologic malignancies. circRNAs have the potential to become new diagnostic markers and therapeutic targets for various diseases. Although further research on circRNAs is still required, circRNA-based therapeutic approaches may be a promising strategy for the treatment of hematologic malignancies in the future.

## Author Contributions

JD designed the study and wrote the manuscript. LW edited and revised the manuscript. All authors have read and approved the submitted version.

## Funding

This work was supported by grants from the key research project program of Shandong Province (2018GSF118035), the Medical Health Science and Technology Development Plan of Shandong Province (2017–462), the Affiliated Hospital Development Fund of Xuzhou Medical University (XYFM2020016).

## Conflict of Interest

The authors declare that the research was conducted in the absence of any commercial or financial relationships that could be construed as a potential conflict of interest.

## Publisher’s Note

All claims expressed in this article are solely those of the authors and do not necessarily represent those of their affiliated organizations, or those of the publisher, the editors and the reviewers. Any product that may be evaluated in this article, or claim that may be made by its manufacturer, is not guaranteed or endorsed by the publisher.

## Abbreviations

circRNAs, circular RNAs; miRNA, microRNA; ecircRNAs, exonic circRNAs; ciRNAs, circular intron RNAs; EIciRNAs, exon-intron circRNAs; tricRNAs, tRNA intronic circular RNAs; RBPs, RNA binding proteins; m6A, N6-methyladenosine; CIRS-7,circRNA sponge for miR-7;IRES,internal ribosome entry site; f-circRNAs, fusion-circular RNAs; AML, Acute myelogenous leukemia; FAB, French–American–British; LFS, leukemia-free survival; *PRKACB, protein kinase CAMP-activated catalytic subunit beta*; FOXM1,forkhead box M1;FLT3,FMS-like tyrosine kinase 3 receptor;PTEN, phosphatase and tensin homolog deleted on chromosome 10; ALL, Acute lymphoblastic leukemia;PVT1, *plasmacytoma variant translocation* 1;CML, Chronic myelogenous leukemia; BCR, breakpoint cluster region; ABL, Abelson;CLL Chronic lymphocytic leukemia; FP-circRNAs,fusion gene chaperone circRNAs; T-LBL, T-cell lymphoblastic lymphoma; DLBCL, Diffuse large B-cell lymphoma; HMGB, high mobility group box; circCDYL, circ-chromodomain Y-like;MCL, mantle cell lymphoma; MM, Multiple myeloma; BTZ, Bortezomib; ISS, International Staging System; circITCH, circRNA itchy E3 ubiquitin protein ligase; IMids, immunomodulatory drugs; PFS, progression free survival; BRD4,bromodomain containing 4; MSCs, mesenchymal stromal cells; ABCG2,ATP-binding cassette transporter G2; MAPK4,mitogen-activated protein kinase 4; β2-MG,beta-2-microglobulin; CR, complete response; ET, essential thrombocythemia.
